# Syntheses, Structures and Properties of Alkali and Alkaline Earth Metal Diamond-Like Compounds Li_2_MgMSe_4_ (M = Ge, Sn)

**DOI:** 10.3390/ma14206166

**Published:** 2021-10-18

**Authors:** Hongbo Gao, Kewang Zhang, Ailijiang Abudurusuli, Chen Bai, Zhihua Yang, Kangrong Lai, Junjie Li, Shilie Pan

**Affiliations:** 1 CAS Key Laboratory of Functional Materials and Devices for Special Environments, Xinjiang Technical Institute of Physics & Chemistry, CAS, Xinjiang Key Laboratory of Electronic Information Materials and Devices, 40-1 South Beijing Road, Urumqi 830011, China; hbgao21@gia.cas.cn (H.G.); zhangkewang@stu.xju.edu.cn (K.Z.); ailijiang18@mails.ucas.ac.cn (A.A.); baichen20@ms.xjb.ac.cn (C.B.); zhyang@ms.xjb.ac.cn (Z.Y.); 2 Department of Physics, Changji University, Changji 831100, China; laikr0212@cjc.edu.cn; 3 College of Physical Science and Technology, Xinjiang University, Urumqi 830046, China; 4 Center of Materials Science and Optoelectronics Engineering, University of Chinese Academy of Sciences, Beijing 100049, China

**Keywords:** diamond-like structure, chalcogenides, infrared nonlinear optical materials, second harmonic generation

## Abstract

Two new diamond-like (DL) chalcogenides, Li_2_MgGeSe_4_ and Li_2_MgSnSe_4_, have been successfully synthesized using a conventional high-temperature solid-state method. The two compounds crystallize in the non-centrosymmetric space group *Pmn*2_1_ with *a* = 8.402 (14) Å, *b* = 7.181 (12) Å, *c* = 6.728 (11) Å, *Z* = 2 for Li_2_MgSnSe_4_, and *a* = 8.2961 (7) Å, *b* = 7.0069 (5) Å, *c* = 6.6116 (6) Å, *Z* = 2 for Li_2_MgGeSe_4_. The calculated results show that the second harmonic generation (SHG) coefficients of Li_2_MgSnSe_4_ (*d*_33_ = 12.19 pm/v) and Li_2_MgGeSe_4_ (*d*_33_ = −14.77 pm/v), mainly deriving from the [MSe_4_] (M = Ge, Sn) tetrahedral units, are close to the one in the benchmark AgGaS_2_ (*d*_14_ = 13.7 pm/V). The calculated band gaps for Li_2_MgSnSe_4_ and Li_2_MgGeSe_4_ are 2.42 and 2.44 eV, respectively. Moreover, the two compounds are the first series of alkali and alkaline-earth metal DL compounds in the I_2_-II-IV-VI_4_ family, enriching the structural diversity of DL compounds.

## 1. Introduction

The exploration of advanced functional materials, as well as the development of structural chemistry, depends on the fabrication of new compounds with a special crystal structure, which contains distinctive physical and chemical behaviors [1,2,3,4,5,6,7,8,9,10,11,12]. A diamond-like (DL) structure compound, exhibiting abundant chemical diversities and adjustable optical properties, has been proven as a valid structural framework for the design and fabrication of new infrared (IR) optical materials, especially for the mid- or far-IR nonlinear optical (NLO) materials. Over the past few decades, a large number of non-centrosymmetric DL chalcogenide compounds, such as Li_4_HgGe_2_S_7_ [13] and Li_4_MgGe_2_S_7_ [14] in the I_4_-II-IV_2_-VI_7_ family, and Li_2_CdGeS_4_ [15], Li_2_CdGeSe_4_ [16], Li_2_ZnGeSe_4_ [17] and Cu_2_ZnSnS_4_ [18] in the I_2_-II-IV-VI_4_ family, with outstanding optical properties, have been developed using an atomic substitution or co-substitution strategy. 

In a DL compound, the cation is coordinated with four anions, and follows the Pauling’s electrostatic valency rule [19,20,21,22,23]. Hence, the optical properties including band gap and SHG response in the DL chalcogenide compounds could be effectively regulated by organizing proper tetrahedral units in the structure. On the basis of the statistical analyses, the DL chalcogenide compounds mainly consisted of univalent metal tetrahedral units, such as alkali metal tetrahedral LiQ_4_ (Q = S, Se) and/or IB group metal tetrahedral M^I^Q_4_ (M^I^ = Cu, Ag; Q = S, Se), with IIB (Zn, Cd and Hg), IIIA (B, Al, Ga and In), IVA (Si, Ge and Sn) and VA (P and As) group element tetrahedral units [24,25,26,27]. Most recently, Pan and Li et al. [14] demonstrated that the alkaline-earth metal AQ_4_ (A = Be, Mg; Q = S, Se) tetrahedral units, which without *d*-*d* and *f*-*f* electronic transitions, can be used to regulate the optical properties of DL chalcogenide compounds. By introducing alkaline-earth metal tetrahedral unit MgS_4_ into the I_4_-II-IV_2_-Q_7_ system, the first alkali and alkaline-earth metal DL sulfide Li_4_MgGe_2_S_7_ with excellent IR NLO optical performances was discovered. However, owing to the experimental challenges to obtain the four-coordinated alkaline-earth metal AQ_4_ tetrahedral units in a crystal structure, the number of reported alkaline-earth metal containing DL compounds is very limited, and the exploration of new IR NLO materials, especially with excellent optical properties in alkali and alkaline-earth metal DL chalcogenide compounds, is just in the initial stage. 

Considering the above discussions, the alkali metal tetrahedral LiS_4_ and alkaline-earth metal tetrahedral MgSe_4_ units were successfully introduced into the classical I_2_-II-IV-VI_4_ family in this work. Two new alkali and alkaline-earth metal DL selenides Li_2_MgMSe_4_ (M = Ge, Sn) were synthesized by conventional high temperature solid state reactions in sealed quartz tubes. Li_2_MgMSe_4_ (M = Ge, Sn) are isostructural compounds, crystallizing in the orthorhombic *Pmn*2_1_ space group. The compounds exhibit a three dimensional channel structure, which is built by [LiSe_4_], [(Li/Mg)Se_4_] and [MSe_4_] (M = Ge, Sn) tetrahedral units. The theoretical investigations show that the calculated band gap for the two compounds is 2.44 eV for Li_2_MgGeSe_4_, and 2.42 eV for Li_2_MgSnSe_4_ (matched with the experimental value of 2.62 eV). The calculated SHG coefficients of the title compounds are *d*_33_ = 12.19 pm/V for Li_2_MgSnSe_4_ and *d*_33_ = −14.77 pm/V for Li_2_MgGeSe_4_, which are close to the one in AgGaS_2_ (*d*_14_ = 13.7 pm/V) [28]. The SHG coefficients are mainly contributed by the MSe_4_ (M = Ge, Sn) tetrahedral units. Meanwhile, the calculated birefringences are 0.011 for Li_2_MgSnSe_4_ and 0.012 for Li_2_MgGeSe_4_. 

## 2. Experimental Sections

### 2.1. Chemical Syntheses

High purity (99.99%) raw materials (Li, Mg, Sn, Ge and Se) were obtained from Aladdin Industrial Corporation (Fengxian District, Shanghai, China) and utilized without extra purification. 

Li_2_MgMSe_4_ (M = Ge, Sn) single crystals for structural determination were prepared using a melting method in sealed quartz tubes. The starting mixture samples (Li:Mg:Ge:Se = 2:1:1:4; Li:Mg:Sn:Se = 2:1:1:4) were packaged in graphite crucibles in a glove box. After that the graphite crucibles were moved into quartz tubes, and the quartz tubes were sealed by flame under a vacuum atmosphere (about 10^−3^ Pa). Then, the samples were heated to 880 °C in 46 h, and kept at 880 °C for 50 h, then cooled to room temperature in 48 h. Breaking the tubes, the yellow Li_2_MgGeSe_4_ and Li_2_MgSnSe_4_ single crystals were harvested in the graphite crucibles. It is worth mentioning that the two crystals show strong moisture absorptions in air.

The syntheses of Li_2_MgMSe_4_ (M = Ge, Sn) powder samples for performance characterization were tried at a higher temperature. The mixtures of Li, Mg, Ge/Sn and Se elements with an atomic stoichiometric ratio were first weighed, ground and sealed in quartz tubes. The sealed samples were slowly heated to 900 °C (in 60 h) in a muffle furnace, and kept at this temperature for 100 h, then cooled to room temperature in 100 h.

### 2.2. Single-Crystal X-ray Diffractions

Li_2_MgMSe_4_ (M = Ge, Sn) single crystals were manually picked out and utilized for structural determinations. The X-ray diffraction data of Li_2_MgMSe_4_ (M = Ge, Sn) single crystals were collected in a Bruker D8 Venture diffractometer that was equipped with monochromatic Mo-K*α* radiation (*λ* = 0.71073 Å) operating at 50 kV and 40 mA. The structure refinements of the two compounds were carried out in the SHELX-97 crystallography software package. The XPREP program was used for the absorption correction (multiscan), the structures of Li_2_MgMSe_4_ (M = Sn, Ge) were checked by PLATON in case of additional symmetry elements [29,30,31]. The detailed processes can be found in previous works [9,14,32,33]. It is worth noting that the initial Li/Mg occupation from refinement was 0.53715:0.462850 for Li_2_MgSnSe_4_, and 0.48933:0.51067 for Li_2_MgGeSe_4_, which is close to 1:1. To maintain the charge balance in the whole structures, the atomic ratio of Li/Mg in both title compounds was set to 1:1. The crystal data and structural refinements of Li_2_MgMSe_4_ (M = Ge, Sn) are listed in Table 1. Meanwhile, the corresponding atomic coordinates, bond distances and angles, isotropic displacement parameters and atomic parameters are shown in Tables S1–S7. Since Li_2_MgGeSe_4_ deliquesces quickly in air, the data collection for Li_2_MgGeSe_4_ was repeated several times using different single crystals. However, the data integrity of Li_2_MgGeSe_4_ is still lower than Li_2_MgSnSe_4_.

### 2.3. Powder X-ray Diffraction (PXRD)

The Powder X-ray diffraction (PXRD) pattern of Li_2_MgSnSe_4_ was characterized using a Bruker D2 Phaser diffractometer (Bruker Corporation, Karlsruhe, Germany) under Cu-Kα radiation (*λ* = 1.5418 Å) with a metal holder. Meanwhile, the experimental XRD pattern of Li_2_MgSnSe_4_ (Figure S1) was recorded from 10 to 70° (2*θ*) with a scan step width of 0.02°. The experimental and calculated PXRD patterns of Li_2_MgSnSe_4_ are shown in Figure S1. Owing to the experimental challenge in synthesizing and characterizing the moisture-sensitive compounds, impurities such as SnSe_2_ and SnSe were observed in the synthesized Li_2_MgSnSe_4_ powder samples. However, based on the XRD patterns, the main phase can be determined to be Li_2_MgSnSe_4_. Meanwhile, compared with Li_2_MgSnSe_4_, Li_2_MgGeSe_4_ powder samples exhibit more serious moisture absorption. It was deliquesced too fast in air (the samples were deliquesced in 1 min at room temperature) to finish the PXRD measurement. GSAS was used to fit and refine the powder diffraction data of Li_2_MgSnSe_4_. The main phase Li_2_MgSnSe_4_ and impurity phases SnSe and SnSe_2_ were refined. A certain peak function was fitted with experimental intensity data, and the values of peaks and structural parameters (including background function, lattice parameters, peak parameters, atomic position, preference orientation, etc.) were constantly adjusted during the fitting process until the difference between calculated intensity and experimental intensity stabilized [34]. The multi-phase Rietveld refinement yielded tiny impurities contents such as SnSe_2_ and SnSe (total 9.7%) remaining from the staring materials, and a weight fraction of 90.3% of target Li_2_MgSnSe_4_ (Figure S2). The refined structural parameters are provided in Table S8. The large difference in the refinement can be attributed to the experimental challenge to obtain long time and high quality PXRD data for the moisture-sensitive Li_2_MgSnSe_4_. However, the refined results are helpful in judging the purity of the product.

### 2.4. UV–Vis–NIR Diffuse Reflectance Spectroscopy

The diffuse reflectance spectrum of the synthesized Li_2_MgSnSe_4_ powder samples was characterized using a DUV spectrophotometer (Shimadzu SolidSpec-3700, Shimadzu Corporation, Shanghai, China) at room temperature in air. Based on the reflection spectrum, the corresponding absorption spectrum was obtained using the Kubelka–Munk formula [35,36]. The process was completed in 5–10 min.

### 2.5. Raman Spectroscopy

The Raman spectrum of Li_2_MgSnSe_4_ was characterized on a single crystal in a LABRAM HR Evolution spectrometer. 

The Li_2_MgSnSe_4_ single crystal was firstly placed onto a transparent glass slide. Then, a suitable objective lens was used to select the measured area on the crystal. The maximum power of the used laser beam was about 60 mW with a spot size of ~35 μm. 

### 2.6. Theoretical Calculations

Based on the density functional theory (DFT) and CASTEP program, the plane wave pseudopotential was applied to calculate the electronic structures of Li_2_MgMSe_4_ (M = Ge, Sn) [37]. Meanwhile, the exchange-correlation effects of the compounds were analyzed by using the generalized gradient approximation (GGA) with the Perdew–Burke–Ernzerhof (PBE) function [38,39]. Under the norm conserving pseudopotentials for wave function expansion, the kinetic energy cutoff of the models was set to 450 eV. Moreover, the Brillouin zone [40] contained 2 × 2 × 2 Monkhorst-pack k-point sampling [41]. The virtual unit cells were used to process the occupancy [42,43].

## 3. Results and Discussion

### 3.1. Crystal Structure

**Figure 1:**
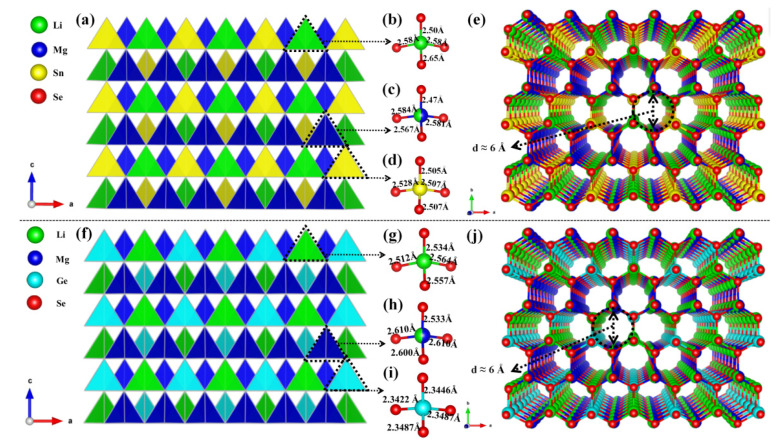
The DL structure of Li_2_MgSnSe_4_ (**a**) and Li_2_MgGeSe_4_ (**f**) on the *ac* plane; (**b**–**d**,**g**–**i**) The structures of [LiSe_4_], [(Li/Mg)Se_4_], [SnSe_4_] and [GeSe_4_] tetrahedral units. The channel-like structures of Li_2_MgSnSe_4_ (**e**) and Li_2_MgGeSe_4_ (**j**) on *ab* plane.

As shown in Figure 1a,f, the two compounds are isomorphic structures. Herein, Li_2_MgSnSe_4_ is taken as an example of the structure description. Li_2_MgSnSe_4_ crystallizes in the noncentrosymmetric space group *Pmn*2_1_ with *a* = 8.402 (14) Å, *b* = 7.181 (12) Å, *c* = 6.728 (11) Å and Z = 2. In the asymmetric unit of Li_2_MgSnSe_4_, there are two Li, one Mg, one Sn and three Se atoms that are crystallographically independent. In Li_2_MgSnSe_4_, the Li2 and Sn1 atoms are bonded to four Se atoms to build up the [LiSe_4_] and [SnSe_4_] tetrahedra with Li-Se bond lengths ranging from 2.50 Å–2.65 Å and Sn-Se bond lengths ranging from 2.505 Å–2.528 Å, respectively. The Li1 and Mg1 atoms are set to share the same sites with the atomic ratio of 1:1 in the initial refinements with the identical anisotropic displacement parameters, which can help to obtain better R values and reasonable temperature factors, similar to the situation of Cu/Mg atomic co-occupation in Cu_2_MgSiS_4_ [44], Cu_2_MgGeS_4_ [44] and Cu_2_MgSiSe_4_ [44]. Furthermore, Li/Mg atomic co-occupation is very common, which can be found in the LiMg(IO_3_)_3_ [45] and Li_0.8_Mg_2.1_B_2_O_5_F [46]. Similar to the Li2 and Sn1 atoms, the co-occupied Li1 and Mg1 atoms are bonded to four Se atoms to construct the [(Li/Mg)Se_4_] tetrahedra units at the Wyckoff position 4b (Table S7). Furthermore, the formed tetrahedra groups are connected with each other by sharing Se atoms to constitute the final DL structure. For both compounds, there is a similar channel-like structure with a channel diameter of about 6 Ångstrom on the *ab* plane, as shown in Figure 1e,j. On the basis of the detailed investigations in the Inorganic Crystal Structure Database (ICSD), the two compounds should be the first series of alkali and alkaline earth metal DL compounds in the I_2_-II-IV-VI_4_ family.

### 3.2. Optical Properties

**Figure 2:**
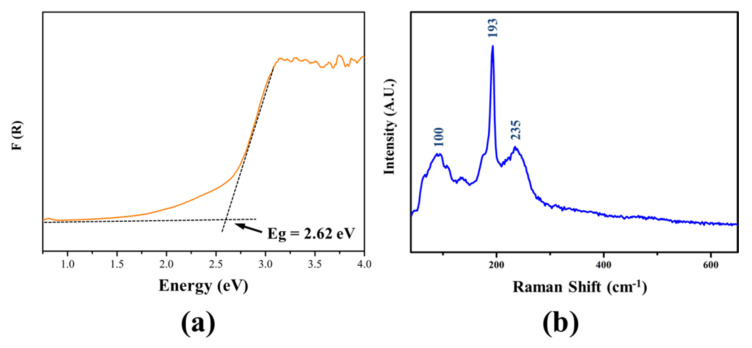
(**a**) Experimental band gap of Li_2_MgSnSe_4_ samples; and (**b**) Raman spectrum of Li_2_MgSnSe_4_ single crystal.

Based on the UV–Vis–NIR diffuse-reflectance spectrum, the experimental band gap of Li_2_MgSnSe_4_ was determined to be 2.62 eV (Figure 2a). To confirm chemical bonding, the Raman spectrum of Li_2_MgSnSe_4_ was characterized on a single crystal. As shown in Figure 2b, the peaks below 193 cm^−1^ are related to the vibrations of Li-Se and Mg-Se bonding, matched with the previous results [47,48,49]. The peak at 193 cm^−1^ and the overlapping peaks around 235 cm^−1^ could be assigned to the asymmetric and symmetric stretching vibrations of Sn-Se bonding in SnSe_4_ tetrahedral groups [49,50]. 

### 3.3. Theoretical Calculations

To study the linear and nonlinear optical properties of Li_2_MgMSe_4_ (M = Ge, Sn), DFT calculations were implemented. Considering the Li/Mg atomic co-occupation at the Wyckoff position 4b in the structures, the virtual unit cells were built for the calculations, as shown in Table S9 and Figure S3. The calculated theoretical band gaps, SHG coefficients and birefringences of the two compounds are shown in Table 2; the calculated band gap for the two compounds is 2.44 eV for Li_2_MgGeSe_4_, and 2.42 eV for Li_2_MgSnSe_4_ (matched with the experimental value of 2.62 eV). The SHG coefficients of Li_2_MgSnSe_4_ in *d*_33_ = 12.19 pm/v and Li_2_MgGeSe_4_ in *d*_33_ = −14.77 pm/v are close to the one of AgGaS_2_ in *d*_14_ = 13.7 pm/v. The calculated birefringences for the two compounds are 0.011 (Li_2_MgSnSe_4_) and 0.012 (Li_2_MgGeSe_4_), respectively. 

**Figure 3:**
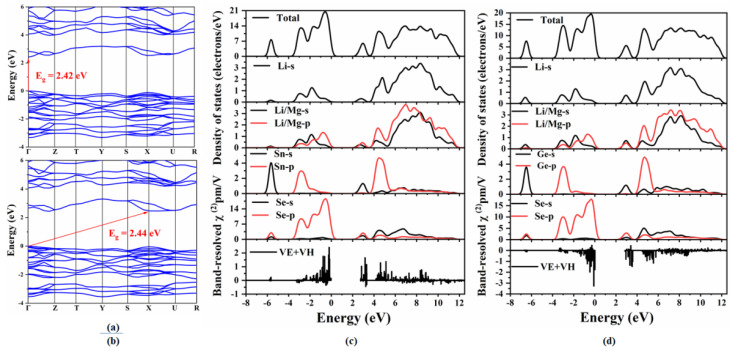
Calculated band structures of (**a**) Li_2_MgSnSe_4_ and (**b**) Li_2_MgGeSe_4_; total and partial density of states and the band-resolved NLO susceptibility χ^(2)^ of (**c**) Li_2_MgSnSe_4_ and (**d**) Li_2_MgGeSe_4_.

To detect the origin of the optical properties, the electronic structures, SHG densities and band-resolved NLO susceptibilities of Li_2_MgMSe_4_ (M = Ge, Sn) were further investigated. Figure 3 shows the calculated band structures, total and partial density of states and the band-resolved NLO susceptibility χ^(2)^ of the two compounds. The band structures (Figure 3a,b) indicate that Li_2_MgGeSe_4_ is an indirect band gap compound with a band gap of 2.44 eV, while Li_2_MgSnSe_4_ is a direct band gap compound with a band gap of 2.42 eV (matched with the experimental value of 2.62 eV). Furthermore, as shown from the total and partial density of states (PDOS) curves (Figure 3c,d), the valence bands maximum (VBM), which, around the Fermi level, is mainly occupied by Se-4*p* (83%) orbitals with the minor contribution of Sn-5*p* (8%), Li/Mg-2*p* (5%) and Li-2*s* (4%) orbitals for Li_2_MgSnSe_4_ and Se-4*p* (83%) orbitals with the minor contribution of Ge-4p (8%), Li/Mg-2*p* (5%) and Li-2*s* (4%) orbitals for Li_2_MgGeSe_4_, respectively (the range from -4.0 to 0 eV). The conduction bands minimum (CBM) originates from Se-4*p* (10%), Sn-5*p* (9%), Li-2*s* (19%) and Li/Mg-2*p* (21%) orbitals for Li_2_MgSnSe_4_, Se-4*p* (11%), Ge-4*p* (8%), Li-2*s* (17%) and Li/Mg-2*p* (21%) orbitals for Li_2_MgGeSe_4_, respectively (the range from 2.4 to 12 eV). The results indicate that the optical band gaps of Li_2_MgMSe_4_ (M = Ge, Sn) are mainly determined by the Se-4*p*, Li/Mg-2*p* and Li-2*s* orbitals. 

**Figure 4:**
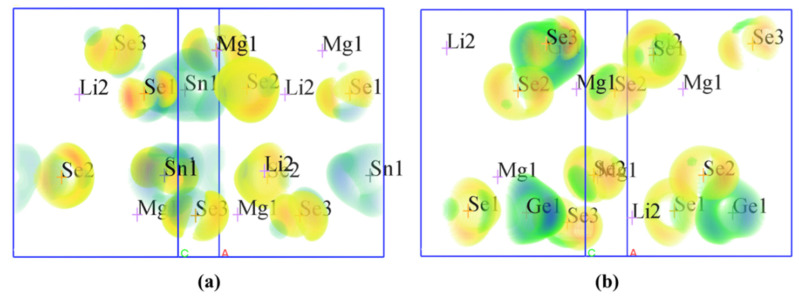
SHG densities of (**a**) Li_2_MgSnSe_4_ and (**b**) Li_2_MgGeSe_4_.

Figure 4 shows the calculated SHG densities for the two compounds. Combined with the band-resolved NLO susceptibility χ^(2)^ in Figure 3c–d, the SHG responses of Li_2_MgMSe_4_ (M = Ge, Sn) can be mainly derived from the [MSe_4_] (M = Ge, Sn) tetrahedra units and with minor contributions from [LiSe_4_] and [(Li/Mg)Se_4_] groups.

## 4. Conclusions

In summary, the first series of DL selenides in the I_2_-II-IV-VI_4_ family, Li_2_MgGeSe_4_ and Li_2_MgSnSe_4_, have been rationally designed and synthesized. Their crystal structures were determined using single crystal X-ray diffractions, and the optical properties were studied using experimental spectra and DFT calculations. Li_2_MgMSe_4_ (M = Ge, Sn) crystallize in the non-centrosymmetric space group *Pmn*2_1_ and show channel structures built by [LiSe_4_], [(Li/Mg)Se_4_] and [MSe_4_] (M = Ge, Sn) tetrahedra units. The two compounds exhibit large theoretical SHG coefficients in *d*_33_ (12.19 pm/v for Li_2_MgSnSe_4_, and −14.77 pm/v for Li_2_MgGeSe_4_), moderate band gaps (2.42 for Li_2_MgSnSe_4_, and 2.44 for Li_2_MgGeSe_4_) in selenides. The results demonstrated that introducing alkali metal and alkaline earth metal tetrahedral units into the I_2_-II-IV-VI_4_ family is a feasible way for the development of diamond-like IR nonlinear optical materials with good properties.

## Figures and Tables

**Table 1:** Crystal data and structural refinements of Li_2_MgSnSe_4_ and Li_2_MgGeSe_4_.

Empirical Formula	Li_2_MgSnSe_4_	Li_2_MgGeSe_4_
Formula weight	472.72 g/mol	426.62 g/mol
Temperature	296.15 K	153 (2) K
Crystal system	Orthorhombic	Orthorhombic
Space group	*Pmn*2_1_ (No. 31)	*Pmn*2_1_ (No. 31)
Unit cell dimensions	*a* = 8.402 (14) Å *b* = 7.181 (12) Å *c* = 6.728 (11) Å	*a* = 8.2961 (7) Å *b* = 7.0069 (5) Å *c* = 6.6116 (6) Å
Volume	405.9 (12) Å^3^	384.33 (5) Å^3^
Z	2	2
Calculated density	3.867 g/cm^3^	3.686 g/cm^3^
Absorption coefficient	21.047 mm^−1^	22.891 mm^−1^
Goodness-of-fit on *F*^2^	0.993	1.110
Final *R* indices [*F*_o_^2^ > 2σ(*F*_o_^2^)] *^[a]^*	*R*_1_ = 0.0349; *wR*_2_ = 0.0750	*R*_1_ = 0.0350; *wR*_2_ = 0.0783
*R* indices	*R*_1_ = 0.0401; *wR*_2_ = 0.0784	*R*_1_ = 0.0413; *wR*_2_ = 0.0831
Largest diff. peak and hole	2.08 e·A^−3^ and −0.93 e·A^−3^	1.55 e·A^−3^ and −2.46 e·A^−3^

*^[a]^ R*_1_ = Σ‖*F*_o_| − |*F*_c_‖/Σ|*F*_o_| and *wR*_2_ = [Σ*w*(*F*_o_^2^ − *F*_c_^2^)^2^/Σ*wF*_o_^4^]^1/2^ for *F*_o_^2^ > 2σ(*F*_o_^2^).

**Table 2:** Calculated band gaps, SHG coefficients and birefringence of Li_2_MgSnSe_4_ and Li_2_MgGeSe_4_.

Compound	Eg (cal./eV)	*d*_15_ (pm/v)	*d*_24_ (pm/v)	*d*_33_ (pm/v)	Δn@1064 nm
Li_2_MgSnSe_4_	2.42	−4.68	−5.81	12.19	0.011
Li_2_MgGeSe_4_	2.4	5.53	7.14	−14.77	0.012

## Data Availability

The data presented in this study are available in Supplementary Material. The X-ray crystallographic coordinates for structures reported in this study have been deposited at the Cambridge Crystallographic Data Centre (CCDC), under deposition numbers 2106592-2106593. These data can be obtained free of charge from The Cambridge Crystallographic Data Centre via www.ccdc.cam.ac.uk/datarequest/cif (accessed on 5 October 2021).

## Supplementary Materials

The following are available online at https://www.mdpi.com/article/10.3390/ma14206166/s1, Table S1: Atomic coordinates and equivalent isotropic displacement parameters of Li_2_MgSnSe_4_, Table S2: Anisotropic displacement parameters (Å^2^ × 10^3^) of Li_2_MgSnSe_4_, Table S3: Symmetry, selected bond lengths and angles of crystal data and structural refinements of Li_2_MgSnSe_4_, Table S4: Atomic coordinates and equivalent isotropic displacement parameters of Li_2_MgGeSe_4_, Table S5: Anisotropic displacement parameters (Å^2^ × 10^3^) of Li_2_MgGeSe_4_, Table S6: Symmetry, selected bond lengths and angles of crystal data and structural refinements of Li_2_MgGeSe_4_, Table S7: Atomic parameters of Li_2_MgSnSe_4_ and Li_2_MgGeSe_4_, Table S8: The refined structural parameters of Li_2_MgSnSe_4_, Table S9: The crystallographic data of Li_2_MgMSe_4_ (M = Sn, Ge), Figure S1: Experimental and calculated PXRD patterns of Li_2_MgSnSe_4_, Figure S2: The PXRD Rietveld refinement of the obtained Li_2_MgSnSe_4_ samples, Figure S3: The atomic models of (a) Li_2_MgSnSe_4_ and (b) Li_2_MgGeSe_4_.
