# Supplementary material for: Syntheses, Structures and Properties of Alkali and Alkaline Earth Metal Diamond-Like Compounds Li2MgMSe4 (M = Ge, Sn)
Source: Materials (Basel). 2021 Oct 18;14(20):6166. doi: 10.3390/ma14206166 (PMC8537313; doi:10.3390/ma14206166)
Supplement: Supplementary file 1 [file materials-14-06166-s001.zip › materials-1382008-supplementary.pdf]

materials MDPI

Supporting Information

Syntheses, Structures and Properties of Alkali and Alkaline Earth Metal Diamond-like Compounds Li_2MgMSe_4 (M = Ge, Sn)

Hongbo Gao [1,2,†], Kewang Zhang [1,3,†], Ailijiang Abudurusuli [1,4], Chen Bai [1], Zhihua Yang [1], Kangrong Lai [2], Junjie Li [1,*] and Shilie Pan [1,*]

[1] CAS Key Laboratory of Functional Materials and Devices for Special Environments, Xinjiang Technical Institute of Physics & Chemistry, CAS; Xinjiang Key Laboratory of Electronic Information Materials and Devices, 40-1 South Beijing Road, Urumqi 830011, China; hbgao21@gia.cas.cn (H.G.); zhangkewang@stu.xju.edu.cn (K.Z.); ailijiang18@mails.ucas.ac.cn (A.A.); baichen20@ms.xjb.ac.cn (C.B.); zhyang@ms.xjb.ac.cn (Z.Y.)
[2] Department of Physics, Changji University, Changji 831100, China; laikr0212@cjc.edu.cn
[3] College of Physical Science and Technology, Xinjiang University, Urumqi 830046, China
[4] Center of Materials Science and Optoelectronics Engineering, University of Chinese Academy of Sciences, Beijing 100049, China
* Correspondence: lijunjie@ms.xjb.ac.cn (J.L.); slpan@ms.xjb.ac.cn (S.P.)
† These authors contributed equally.

* The calculated details for the contribution of each PDOS on the band gaps in $Li_2MgSnSe_4$, $Li_2MgGeSe_4$.

The contribution of each PDOS on the band gaps was calculated from the following formula:

$$\%_{contribution} = \frac{S_{PDOS}}{S_{TDOS}} \quad (S1)$$

Here, *%contribution* is the contribution of each PDOS, $S_{PDOS}/S_{TDOS}$is the integral of each PDOS/TDOS at near the Fermi level.

Table S1. Atomic coordinates and equivalent isotropic displacement parameters of $Li_2MgSnSe_4$. U(eq) is defined as one third of the trace of the orthogonalized U_{ij} tensor.

Atom	x	y	z	U(eq)
**Sn(1)**	0	3280.6 (13)	9172 (15)	15.6 (3)
**Mg(1)**	2440 (6)	8297 (9)	9320 (50)	17 (4)
**Li(1)**	2440 (6)	8297 (9)	9320 (50)	17 (4)
**Li(2)**	0	6540 (30)	4000 (200)	16 (14)
**Se(1)**	0	6574 (2)	7943 (3)	19.1 (4)
**Se(2)**	0	3211 (2)	12929 (3)	18.5 (5)
**Se(3)**	2467.9 (13)	1665.7 (16)	7982.1 (19)	18.4 (4)

Table S2. Anisotropic displacement parameters (Å^2×10^3) of $Li_2MgSnSe_4$.

Atom	U_{11}	U_{22}	U_{33}	U_{23}	U_{13}	U_{12}
**Sn(1)**	17.8 (5)	17.1 (5)	11.9 (7)	0.5 (8)	0	0
**Mg(1)**	24 (4)	22 (4)	6 (11)	3 (5)	4 (5)	-3 (2)
**Li(1)**	24 (4)	22 (4)	6 (11)	3 (5)	4 (5)	-3 (2)
**Li(2)**	27 (13)	13 (12)	10 (40)	0 (20)	0	0
**Se(1)**	20.6 (8)	17.9 (8)	18.6 (11)	3.6 (13)	0	0
**Se(2)**	18.0 (8)	23.5 (8)	14.1 (12)	2.5 (18)	0	0
**Se(3)**	19.8 (6)	21.1 (6)	14.5 (10)	-0.8 (10)	3.1 (6)	2.0 (4)

Table S3. Symmetry, selected bond lengths and angles of crystal data and structural refinements of $Li_2MgSnSe_4$.

**Sn(1)-Se(1)**	2.505 (5)	**Se(1)-Sn(1)-Se(2)**	110.4 (3)
**Sn(1)-Se(2)**	2.528 (11)	**Se(1)-Sn(1)-Se(3)**	109.3 (2)
**Sn(1)-Se(3)#1**	2.507 (4)	**Se(1)-Sn(1)-Se(3)#1**	109.3 (2)
**Sn(1)-Se(3)**	2.507 (4)	**Se(3)-Sn(1)-Se(2)**	108.1 (2)
**Li(1)-Se(1)**	2.567 (14)	**Se(3)#1-Sn(1)-Se(2)**	108.1 (2)
**Li(1)-Se(2)#2**	2.583 (13)	**Se(3)#1-Sn(1)-Se(3)**	111.6 (3)
**Li(1)-Se(3)#3**	2.47 (3)	**Se(1)-Li(1)-Se(2)#2**	109.4 (8)
**Li(1)-Se(3)#4**	2.581 (14)	**Se(1)-Li(1)-Se(3)#3**	109.5 (8)
**Li(2)-Se(1)**	2.65 (16)	**Se(3)#4-Li(1)-Se(1)**	113.0 (7)
**Li(2)-Se(2)#5**	2.50 (4)	**Se(3)#3-Li(1)-Se(2)#2**	105.0 (7)
**Li(2)-Se(3)#2**	2.58 (5)	**Se(3)#4-Li(1)-Se(2)#2**	109.9 (7)
**Li(2)-Se(3)#6**	2.58 (5)	**Se(3)#4-Li(1)-Se(3)#3**	109.7 (7)
		**Se(2)#5-Li(2)-Se(1)**	107 (4)
		**Se(2)#5-Li(2)-Se(3)#2**	114 (3)
		**Se(2)#5-Li(2)-Se(3)#6**	114 (3)
		**Se(3)#2-Li(2)-Se(1)**	105 (3)
		**Se(3)#6-Li(2)-Se(1)**	105 (3)
		**Se(3)#2-Li(2)-Se(3)#6**	111 (3)

#1 −x,+y,+z #2 1/2 − x,1 − y,−1/2 + z #3 +x,1 + y,+z #4 1/2 − x,1 − y,1/2 + z.
#5 +x,+y,−1 + z #6 −1/2 + x,1 − y,−1/2 + z #7 −1/2 + x,1 − y,1/2 + z.
#8 +x,+y,1 + z #9 +x,−1 + y,+z.

Table S4. Atomic coordinates and equivalent isotropic displacement parameters of $Li_2MgGeSe_4$. U(eq) is defined as one third of the trace of the orthogonalized U_{ij} tensor.

Atom	x	y	z	U(eq)
**Ge(1)**	10000	1762.4 (8)	6416.9 (9)	6.43 (11)
**Mg(1)**	7419 (3)	3215 (4)	1428 (6)	10.5 (4)
**Li(1)**	7419 (3)	3215 (4)	1428 (6)	10.5 (4)
**Li(2)**	5000	1560 (20)	6570 (30)	21 (3)
**Se(1)**	10000	1840.6 (8)	2871.6 (8)	7.69 (12)
**Se(2)**	7654.8 (5)	3290.6 (5)	7573.3 (5)	8.93 (9)
**Se(3)**	10000	-1378.9 (8)	7628.3 (11)	8.60 (10)

Table S5. Anisotropic displacement parameters ($Å^2 \times 10^3$) of $Li_2MgGeSe_4$.

Atom	U_{11}	U_{22}	U_{33}	U_{23}	U_{13}	U_{12}
**Ge(1)**	7.3 (2)	5.7 (2)	6.3 (2)	0.14 (16)	0	0
**Mg(1)**	9.7 (11)	10.7 (11)	11.0 (11)	-0.5 (8)	0.7 (10)	0.9 (8)
**Li(1)**	9.7 (11)	10.7 (11)	11.0 (11)	-0.5 (8)	0.7 (10)	0.9 (8)
**Li(2)**	17 (6)	27 (7)	20 (7)	12 (5)	0	0
**Se(1)**	6.00 (18)	11.0 (2)	6.1 (3)	0.52 (17)	0	0
**Se(2)**	9.23 (14)	7.99 (15)	9.6 (2)	-0.58 (17)	2.09 (16)	0.98 (10)
**Se(3)**	9.95 (18)	5.92 (19)	9.9 (2)	0.9 (3)	0	0

Table S6. Symmetry, selected bond lengths and angles of crystal data and structural refinements of $Li_2MgGeSe_4$.

**Ge(1)-Se(1)**	**2.3446 (7)**	**Se(1)-Ge(1)-Se(2)#1**	**108.35 (2)**
**Ge(1)-Se(2)**	2.3487 (5)	**Se(1)-Ge(1)-Se(2)**	108.35 (2)
**Ge (1)-Se(2)#1**	2.3487 (5)	**Se(2)#1-Ge(1)-Se(2)**	111.86 (3)
**Ge (1)-Se(3)**	2.3422 (9)	**Se(3)-Ge(1)-Se(1)**	111.34 (3)
**Li(1)#1-Se(1)**	2.534 (3)	**Se(3)-Ge(1)-Se(2)**	108.49 (2)
**Li(2)#4-Se(1)**	2.533 (14)	**Se(3)-Ge(1)-Se(2)#1**	108.49 (2)
**Li(1)#7-Se(2)**	2.564 (3)	**Se(1)-Mg(1)-Se(2)#2**	105.77 (12)
**Li(1)#8-Se(2)**	2.557 (4)	**Se(1)-Mg(1)-Se(2)#3**	108.58 (11)
**Li(1)#9-Se(3)**	2.512 (3)	**Se(2)#3-Mg(1)-Se(2)#2**	106.03 (11)
**Li(1)#5-Se(3)**	2.512 (3)	**Se(3)#4-Mg(1)-Se(1)**	111.20 (12)
**Li(2)#5-Se(3)**	2.610 (18)	**Se(3)#4-Mg(1)-Se(2)#2**	112.13 (11)
**Li(2)-Se(1)#5**	2.533 (14)	**Se(3)#4-Mg(1)-Se(2)#3**	112.74 (12)
**Li(2)-Se(2)**	2.600 (9)	**Se(1)#5-Li(2)-Se(2)**	110.6 (4)
**Li(2)-Se(2)#6**	2.600 (9)	**Se(1)#5-Li(2)-Se(2)#6**	110.6 (4)
**Li(2)-Se(3)#4**	2.610 (18)	**Se(1)#5-Li(2)-Se(3)#4**	107.0 (6)
		**Se(2)-Li(2)-Se(2)#6**	115.8 (6)
		**Se(2)#6-Li(2)-Se(3)#4**	106.1 (4)
		**Se(2)-Li(2)-Se(3)#4**	106.1 (4)

#1 2 − x, +y, +z #2 3/2 − x,1 − y, −1/2 + z #3 + x, +y, −1 + z #4 3/2 − x, −y, −1/2 + z.
#5 3/2 − x, −y, 1/2 + z #6 1 − x, 1 + y, +z #7 +x, +y, +z.
#8 3/2 − x, 1 − y, 1/2 + z #9 1/2 + x, −y, 1/2 + z.

Table S7. Atomic parameters of $Li_2MgSnSe_4$ and $Li_2MgGeSe_4$.

	**$Li_2MgSnSe_4$**			**$Li_2MgGeSe_4$**	
**Atom**	**Wyckoff Site**	**S.O.F.**	**Atom**	**Wyckoff Site**	**S.O.F.**
**Sn(1)**	2a		**Ge(1)**	2a	
**Mg(1)**	4b	0.5	**Mg(1)**	4b	0.5
**Li(1)**	4b	0.5	**Li(1)**	4b	0.5
**Li(2)**	2a		**Li(2)**	2a	
**Se(1)**	2a		**Se(1)**	2a	
**Se(2)**	2a		**Se(2)**	4b	
**Se(3)**	4b		**Se(3)**	2a	

Table S8. The refined structural parameters of $Li_2MgSnSe_4$.

$Li_2MgSnSe_4$	
a	8.402 Å
b	7.181 Å
c	6.728 Å
α = β = γ	90 °
V **(Å³)**	405.932 Å³
Rwp **(%)**	10.29 %
Rp **(%)**	7.47 %
χ^2	11.70

Table S9. The crystallographic data of Li_2MgMSe_4 (M = Sn, Ge).

**$Li_2MgSnSe_4$**					
***Pmn*2₁ (No.31)**					
**Cell parameters**					
a **(Å)**	***b*** **(Å)**	***c*** **(Å)**	***α*** **(°)**	***β*** **(°)**	***γ*** **(°)**
8.402	7.181	6.728	90	90	90
**Fractional coordinates**					
**Atoms**	**x**		**y**		**z**
Li	0.00000		0.65400		0.40000
Li(0.5)/Mg(0.5)	0.24400		0.82970		0.93200
Sn	0.00000		0.32806		0.91720
Se1	0.00000		0.65740		0.79430
Se2	0.00000		0.32110		1.29290
Se3	0.24679		0.16657		0.79821
**$Li_2MgGeSe_4$**					
***Pmn*2₁ (No.31)**					
**Cell parameters**					
a **(Å)**	***b*** **(Å)**	***c*** **(Å)**	***α*** **(°)**	***β*** **(°)**	***γ*** **(°)**
8.296	7.007	6.612	90	90	90
**Fractional coordinates**					
**Atoms**	**x**		**y**		**z**
Li	0.50000		0.15600		0.65700
Li(0.5)/Mg(0.5)	0.74190		0.32150		0.14280
Ge	1.00000		0.17624		0.64169
Se1	1.00000		0.18406		0.28716
Se2	0.76548		0.32906		0.75733
Se3	1.00000		−0.13789		0.76283

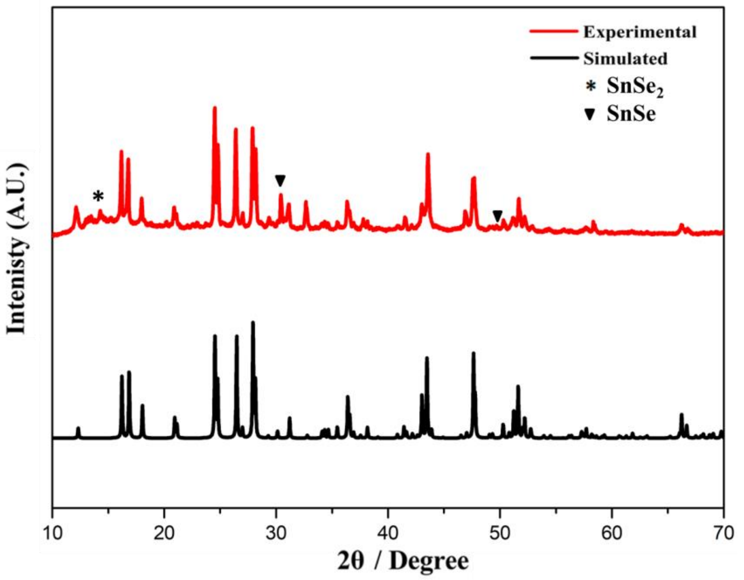

Figure S1. Experimental and calculated PXRD patterns of $Li_2MgSnSe_4$.

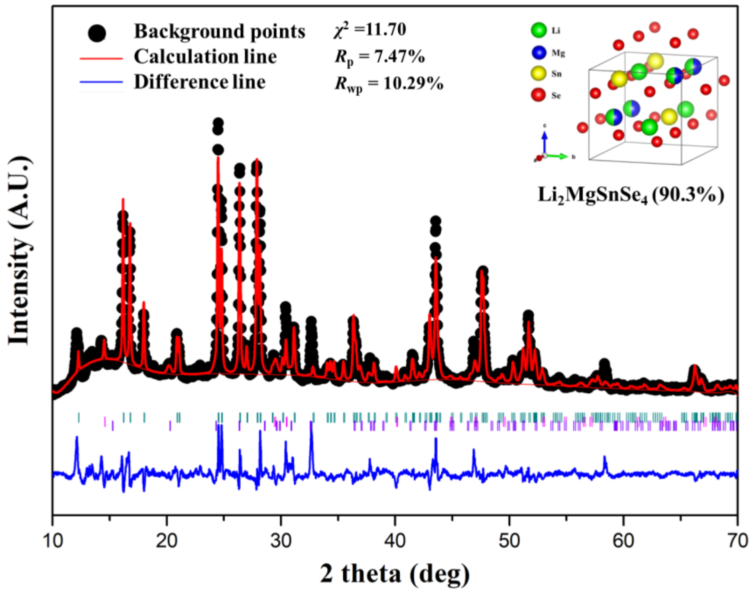

Figure S2. The PXRD Rietveld refinement of the obtained $Li_2MgSnSe_4$ samples.

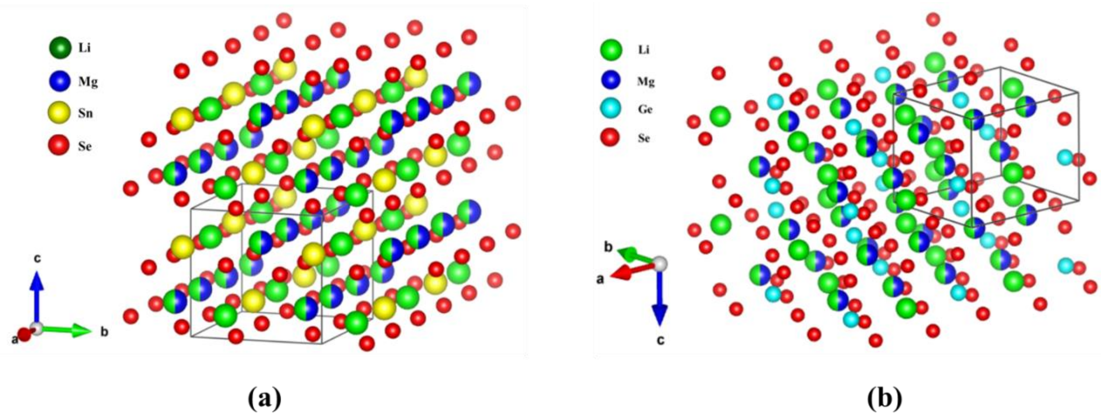

Figure S3. The atomic models of (**a**) $Li_2MgSnSe_4$ and (**b**) $Li_2MgGeSe_4$.
